# Supplementary material for: Estimating total human deaths from rabies: A statistical framework leveraging reports of rabies transmission from deceased organ and tissue donors
Source: PLoS Negl Trop Dis. 2026 Sep 21;20(9):e0014731. doi: 10.1371/journal.pntd.0014731 (PMC13592719; doi:10.1371/journal.pntd.0014731)
Supplement: Supplementary file 6 [file pntd.0014731.s005.zip › Result_A/Bangladesh/Figure_S3_Bangladesh.pdf]

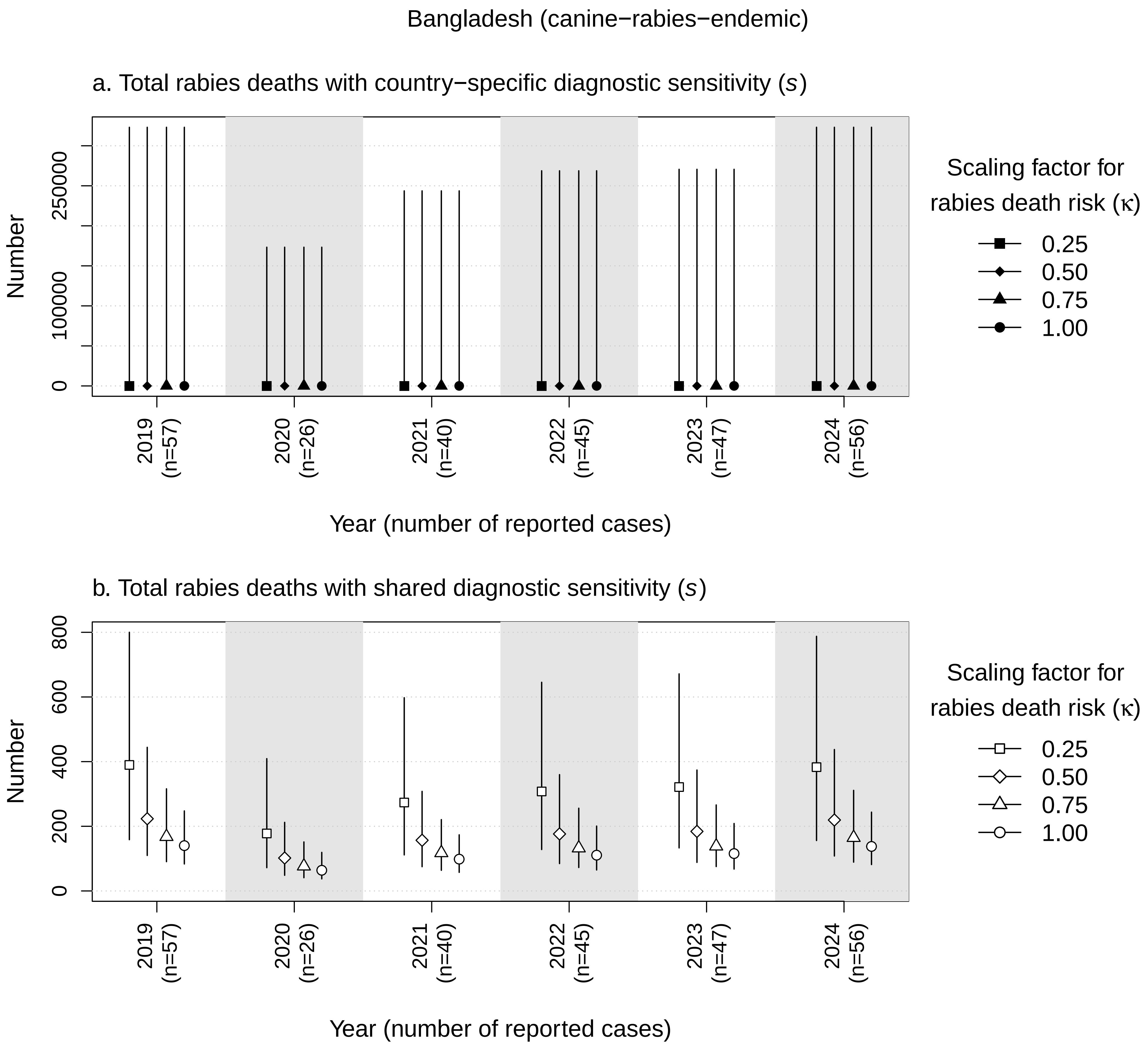

Bangladesh (canine−rabies−endemic)
a. Total rabies deaths with country−specific diagnostic sensitivity (s)
Number
0
100000
250000
2019 (n=57)
2020 (n=26)
2021 (n=40)
2022 (n=45)
2023 (n=47)
2024 (n=56)
Year (number of reported cases)
Scaling factor for rabies death risk (κ)
0.25
0.50
0.75
1.00
b. Total rabies deaths with shared diagnostic sensitivity (s)
Number
0
200
400
600
800
2019 (n=57)
2020 (n=26)
2021 (n=40)
2022 (n=45)
2023 (n=47)
2024 (n=56)
Year (number of reported cases)
Scaling factor for rabies death risk (κ)
0.25
0.50
0.75
1.00
